# Unraveling the therapeutic potential of quercetin and quercetin-3-O-glucuronide in Alzheimer's disease through network pharmacology, molecular docking, and dynamic simulations

**DOI:** 10.1038/s41598-024-61779-9

**Published:** 2024-06-27

**Authors:** Sarvesh Sabarathinam

**Affiliations:** Drug Testing Laboratory, Interdisciplinary Institute of Indian System of Medicine (IIISM), SRM Institute of Science and Technology, Kattankulathur, Chennai, Tamil Nadu 603203 India

**Keywords:** Quercetin, Quercetin-3-O-glucuronide, Alzheimer's disease, Network-pharmacology, Molecular-dynamics, Chemical biology, Computational biology and bioinformatics, Developmental biology, Drug discovery, Chemistry

## Abstract

Quercetin is a flavonoid with notable pharmacological effects and promising therapeutic potential. Quercetin plays a significant role in neuroinflammation, which helps reduce Alzheimer's disease (AD) severity. Quercetin (Q) and quercetin 3-O-glucuronide (Q3OG) are some of the most potent antioxidants available from natural sources. However, the natural form of quercetin converted into Q3OG when reacted with intestinal microbes. The study aims to ensure the therapeutic potential of Q and Q3OG. In this study, potential molecular targets of Q and Q3OG were first identified using the Swiss Target Prediction platform and pathogenic targets of AD were identified using the DisGeNET database. Followed by compound and disease target overlapping, 77 targets were placed in that AKT1, EGFR, MMP9, TNF, PTGS2, MMP2, IGF1R, MCL1, MET and PARP1 was the top-ranked target, which was estimated by CytoHubba plug-in. The Molecular docking was performed for Q and Q3OG towards the PDB:1UNQ target. The binding score of Q and Q3OG was − 6.2 kcal/mol and − 6.58 kcal/mol respectively. Molecular dynamics simulation was conducted for Q and Q3OG towards the PDB:1UNQ target at 200 ns. This study's results help identify the multiple target sites for the bioactive compounds. Thus, synthesizing new chemical entity-based quercetin on structural modification may aid in eradicating AD complications.

## Introduction

Alzheimer's disease (AD) is a neurological condition that remains the **5**^**th**^ leading cause of mortality. Data shows that an estimated 18 billion hours of care were given to people with Alzheimer's or other dementias in 2022 by more than 11 million family members and other unpaid caregivers^1^. Since there is no standard treatment pattern for AD, a nutrition-based approach appears essential in preventing neurodegenerative illnesses. A healthy, bioactive-rich diet can lower the chance of developing dementia^2^. Plants are rich in bioactive compounds like phenols, Glycosides, vitamins, glycosides, anthocyanins, Flavones, alkaloids , tannins etc. "Secondary metabolites" of plants are believed to be naturally occurring bioactive compounds with significant therapeutic value. In this regard, different compounds isolated from various plant parts, such as roots, rhizomes, leaves, and seeds, have been demonstrated to prevent detrimental plaque development and improve cholinergic transmission. Since oxidative stress is the main leading factor in many chronic disorders, there is a demand for Foods’s high in antioxidants. As a result, scientists are interested in plant-derived chemicals since they have various pharmacological effects and can be used to create compounds that can treat multiple diseases.^3^ Traditional systems of medicine, often rooted in plants, have been used for generations to treat various ailments takes a holistic approach to health, focusing on overall well-being rather than just symptom management^4^. This approach aligns with the complex nature of AD. Natural compounds found in plants contain a mixture of compounds that can target multiple pathways involved in AD pathophysiology and might also render a synergistic effect, addressing various aspects of the disease simultaneously^5^. Based on numerous epidemiological studies, it is confirmed that plant-based bioactive compounds, such as polyphenols and terpenoids, are abundant in antioxidant and anti-inflammatory properties and that they appear to favor a delay in the onset of degenerative disorders like AD, dementia in which oxidative stress plays a significant role^6^. Quercetin (Q) and quercetin 3-O-glucuronide (Q3OG) are some of the most potent antioxidants available from natural sources. Q and Q3OG is available in raw form in many plants and vegetables. Neuroinflammation is a distinctive mark in the pathogenesis of AD. While stimulating neuronal regeneration during inflammations, Quercetin suppresses the neuroinflammatory processes by downregulating pro-inflammatory cytokines, such as NF-kB and iNOS. Quercetin counteracts oxidative stress-induced cell damage in neurons at low micromolar doses^7–10^. Quercetin has effects that include inhibiting tau phosphorylation and amyloid beta aggregation, followed by preventing the hydrolysis of acetylcholine by the AChE enzyme, which raises levels of acetylcholine^9^. Hence the present study is aimed to estimate the Network analysis, Molecular docking and dynamic simulation of Q and Q3OG towards Alzheimer’s disease.

## Methods and materials

### Target prediction of Q and Q3OG against AD

The potential targets of Q and Q3OG were predicted using swiss Target Prediction database (http://www.swisstargetprediction.ch/)^11^. The canonical smiles were obtained from PubChem database (https://pubchem.ncbi.nlm.nih.gov/) and were uploaded to the swiss Target Prediction server. Species “Homo sapiens” **were** selected to standardize the uniport ID to the gene symbols. At the same time, the targets of AD were predicted in the DisGeNet database (https://www.disgenet.org/home/)^12^. The keyword “ Alzheimer’s Disease **(C0002395)**” **were** used to collect potential genes. For further analysis was performed by overlapping the AD targets and component targets using the Venn diagram which represented the potential targets of Q and Q3OG against AD.

### Gene ontology and KEGG pathway enrichment analysis

The core mechanism and pathway of Q and Q3OG anti-AD was explored by GO function and KEGG pathway enrichment analysis. We hub genes obtained from the overlapping was searched in the DAVID database (https://david.ncifcrf.gov) by limiting the species to “*Homo sapiens*”. Biological process (BP), cell composition (CC), molecular function (MF), and KEGG pathways were collected^12^.

### Construction of the compound-target network

Cytoscape 3.10.0 (https://cytoscape.org/) was employed for network construction and visualization, allowing analysis of biomolecular interaction networks. In the network representation, nodes represented the active constituents and target genes, while edges depicted the interactions between the active constituents and their target genes^13^.

### Protein-protein interaction (PPI) network construction

The STRING (http://string-db.org; Version 11.5) database was used to construct a protein-protein interaction (PPI) network for Q and Q3OG anti-AD to analyze the functional interaction between proteins. The network confidence score ≥0.4 was set to obtain targets with “*Homo sapiens*” being selected in Cytoscape software (version 3.10.0) further. CytoHubba plug-ins were used to collect the core targets of Q, Q3OG anti-AD. The top-ranked targets were scrutinized using the Cyto-Hubba plug-in Cytoscape software my means of selecting the top 10 Hubba nodes using Maximal Clique Centrality (MCC) ratio.

### Molecular docking

Target protein was obtained by comprehensively evaluating the resolution and release time in the Protein Data Bank (PDB) (www.rcsb.org) website, and chemical structures of active components were downloaded from the PubChem database. The refinement was performed using Chimera software (https://www.cgl.ucsf.edu/chimera/), the docking was performed from the CD DOCK 2 webserver (https://cadd.labshare.cn/cb-dock2/php/index.php)^14^. The structures were generated using Discovery Studio Software. In this study the Molecular docking of Q and Q3OG was carried towards the PDB ID: 1UNQ target (**PDB: 1UNQ is a Non-mutant, Transferase protein with resolution of 0.98Å, which is corresponds to the protein structure of Interleukin-1 beta (IL-1β), which plays a significant role in inflammatory responses)**^15^.

### Molecular dynamic simulation

Molecular dynamics (MD) simulation was carried out using GROMACS 2022.2. The following steps were utilized. The 3-dimensional (3D) models of ligand-protein complexes were exported to .pdb format using Pymol. The dynamic behavior of the complexes was evaluated using molecular dynamic (MD) simulation in the GROMACS package program (version 2022.2)^16,17^. Protein topology was constructed by pdb2gmx with the CHARMM27 force field^18^ and ligand topology was generated using the SwissParam server.^19^ After applying the force field, the complexes were inserted into the system. They were solvated with the TIP3P water model^20^ in a cubic box greater than 1 nm from the edge of the protein with periodic boundary conditions. The system was neutralized by adding Na+ ions, and energy minimization was done for 50,000 steps using the steepest descent algorithm. It was then followed by 100 ps of NVT simulation at 300 K and 100 ps of NPT simulation to equilibrate the whole system. Leapfrog algorithm was employed in the constant-temperature, constant-pressure (NPT) ensemble to separately couple each component like protein, ligand, water molecules, and ions^21^. The Berendsen temperature and pressure coupling constants were set to .1 and 2, respectively, to keep the system in a stable environment (300 K temperature and 1 bar pressure). 9.03×1019 Na+ was added during the molecular dynamic simulations^22^ Finally, MD simulation for 100 ns was performed in isothermal and isobaric condition ensemble at 300 K. The pressure coupling with time-constant was set at 1 ps to maintain pressure constant at 1 bar, and LINCS algorithm was used to constrain the bond lengths. The Van der Waals and Coulomb interactions were truncated at 1.2 nm, and the PME algorithm ^23^ inbuilt in GROMACS was used to minimize the error from truncation. The trajectory files are visualized through VMD (Visual Molecular Dynamics) 1.9.2.^24^ and analyzed by indigenously developed tool HeroMDAnalysis^25,26^ and Xmgrace 5.1.25.^27^

## Results

### Screened Q, Q3OG compounds targets and AD disease targets

Q and Q3OG were screened according to the screening criteria of OB ≥ 30% and DL ≥ 0.18 and the canonical smiles were imported to the swiss target prediction database. Quercetin passes all the rules without violations. Quercetin 3-O-glucuronide has been reported with a minimum of one violation of all the rules”. The drug-likeness profile of Q and Q3OG is given in Table 1. While coming to Pharmacokinetic parameters estimation Q seem to have higher GI absorption and Q3OG seems have low GI absorption. Q is inhibitor of CYP1A2, CYP2D6 and CYP3A4. The pharmacokinetic profile of Q and Q3OG is given in Table 2. A total of 100 targets were obtained where Q and Q3OG had 100 targets each. The targets of Q and Q3OG is given in supplementary file-1 And a total of 3,397 targets of AD were identified from DisGeNET database. Both targets of Q, Q3OG and AD were imported into a Venn diagram and the 77 overlapped genes were the potential targets for Q, Q3OG anti-AD. The hub-genes of the overlapped targets were given in supplementary file-2

**Table 1:** Drug-likeness properties of Q and Q3OG.

Quercetin	Lipinski	Yes; 0 violation
	Ghose	Yes
	Veber	Yes
	Egan	Yes
	Muegge	Yes
Quercetin 3-O-glucuronide	Lipinski	No; 2 violations: NorO>10, NHorOH>5
	Ghose	No; 1 violation: WLOGP<-0.4
	Veber	No; 1 violation: TPSA>140
	Egan	No; 1 violation: TPSA>131.6
	Muegge	No’ 3 violations: TPSA>150, H-acc>10, H-don>5

**Table 2:** Pharmacokinetic properties of Q and Q3OG.

Quercetin	Gl absorption	High
	BBB permeant	No
	P-gp sunstrate	No
	CYP1A2 inhibitor	Yes
	CYP2C19 inhibitor	No
	CYP2C9 inhibitor	No
	CYP2D6 inhibitor	Yes
	CYP3A4 inhibitor	Yes
Q3OG	Gl absorption	Low
	BBB permeant	No
	P-gp sunstrate	Yes
	CYP1A2 inhibitor	No
	CYP2C19 inhibitor	No
	CYP2C9 inhibitor	No
	CYP2D6 inhibitor	No
	CYP3A4 inhibitor	No

### Compound- target network analysis

**Figure 1:**
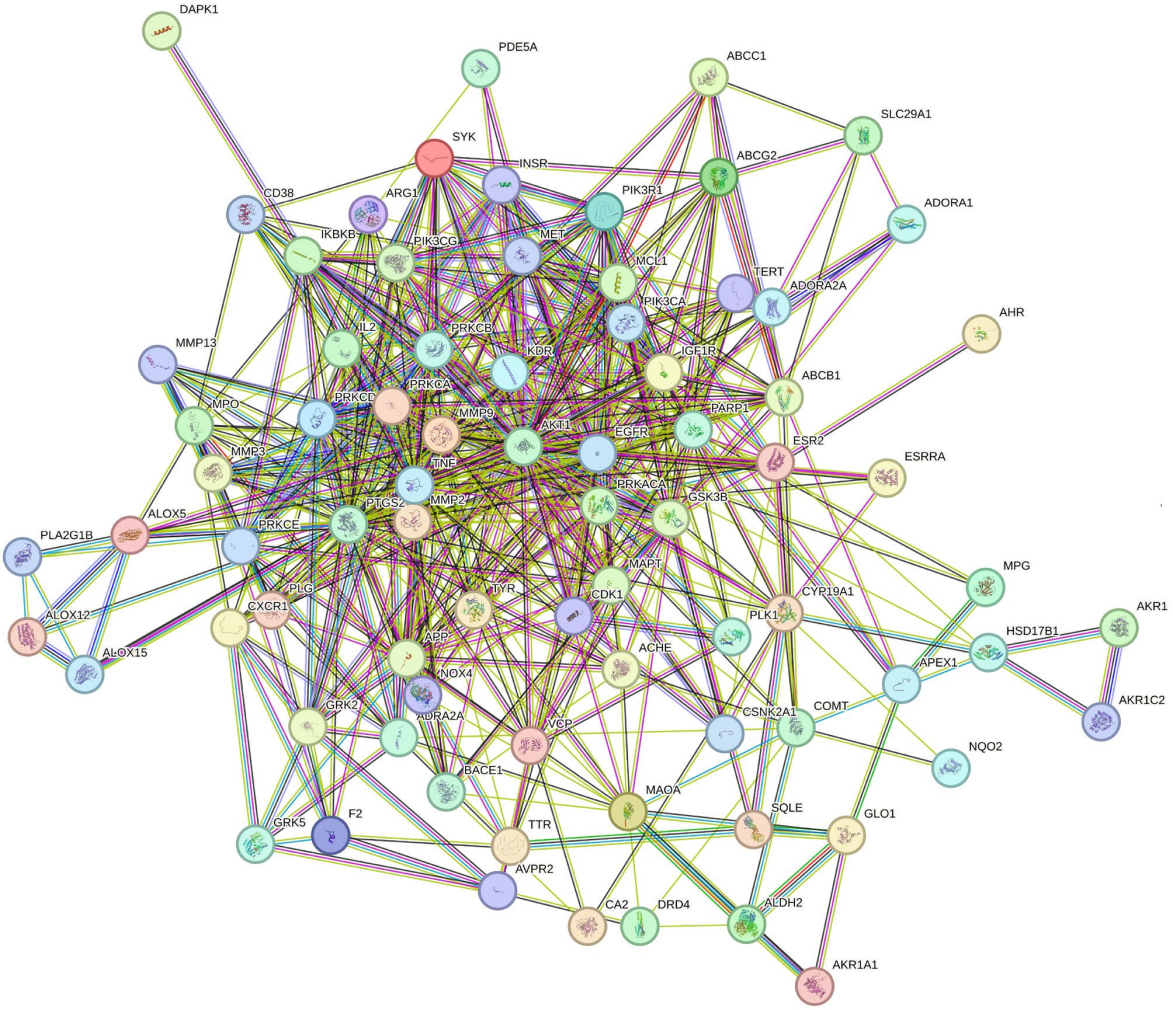
Network analysis of hub-genes.

**Figure 2:**
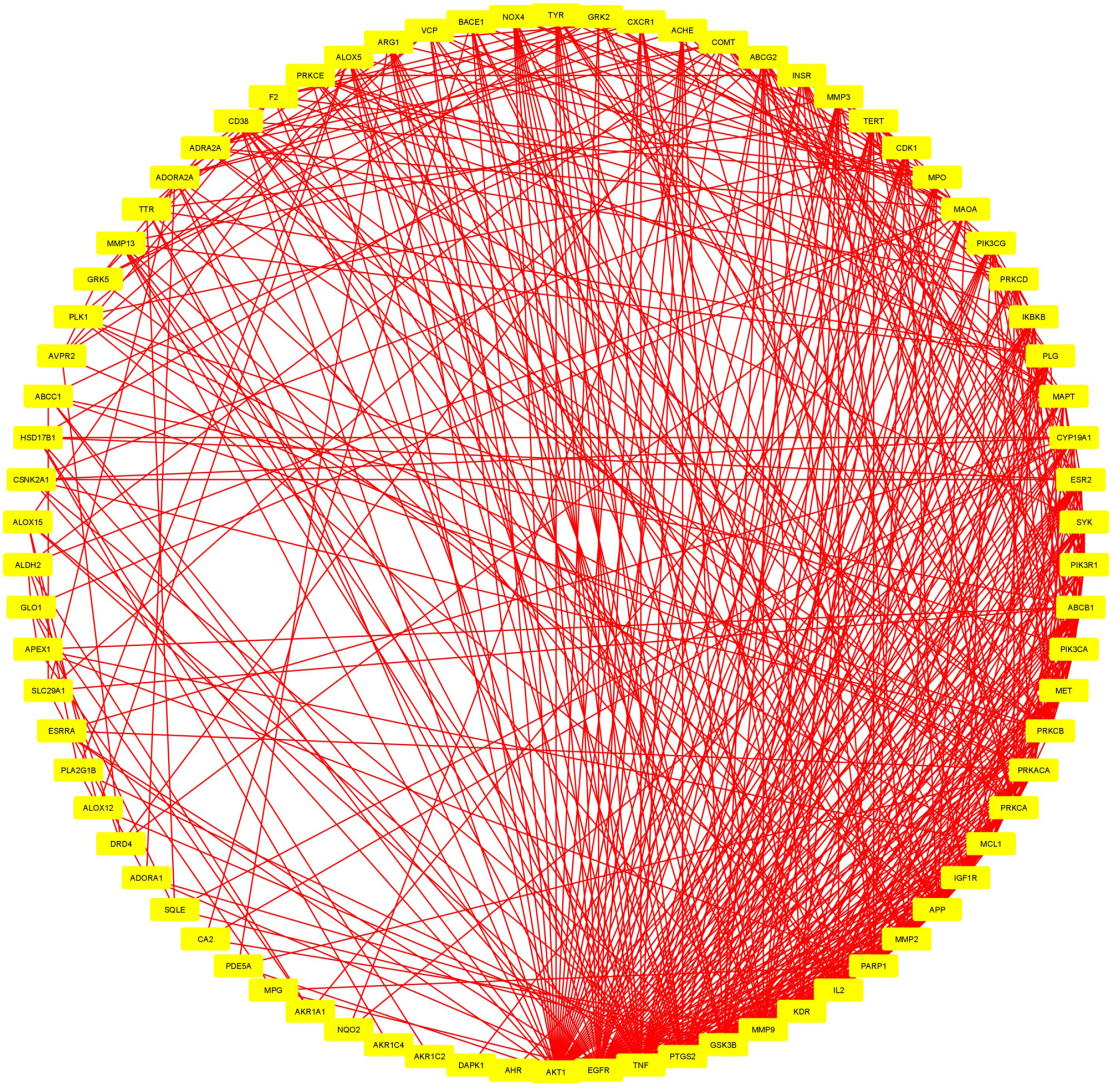
Network construction of hub-genes.

Additionally, Q and Q3OG were selected along with 200 targets, and their corresponding pathways that involve a maximal count of genes. This selection was made to develop a network diagram depicting the connections between active compounds, targeted genes, and pathways was generated using cytoscape. The existence of numerous targets linked to each active compound strongly implies the potential of utilizing these two compounds as agents against AD. This observation underscores that the compounds' impact on multiple targets could synergistically amplify their efficacy in the treatment of AD. The network analysis of hub-genes is illustrated in Figure 1. The network construction of Hub-genes is depicted in Figure 2. The network analysis data of the hub-genes is given supplementary file-3

### Gene-ontology function and KEGG pathway enrichment analysis

**Figure 3:**
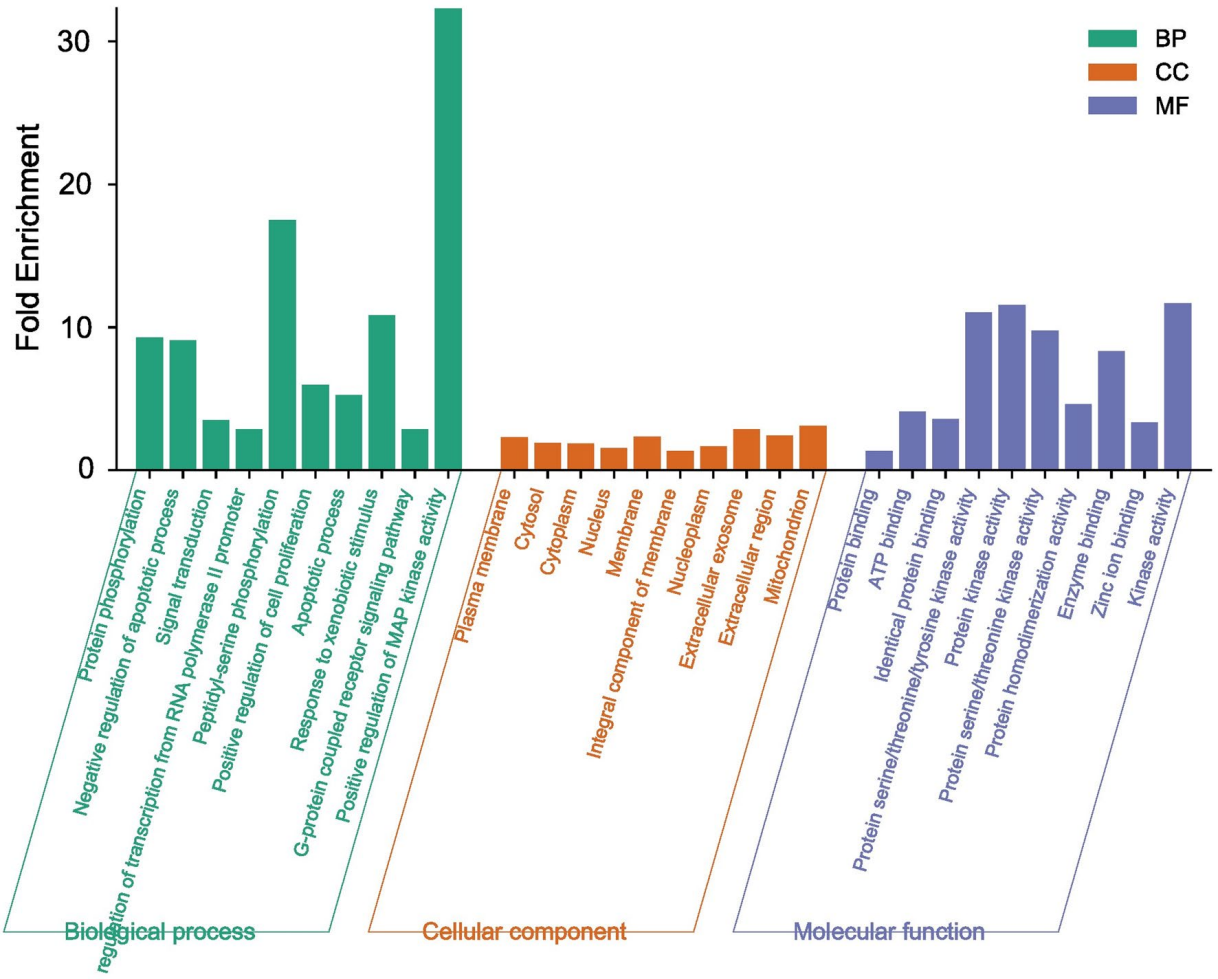
Biological process, Cellular Component ,Molecular Function pathways of the hub-genes.

**Figure 4:**
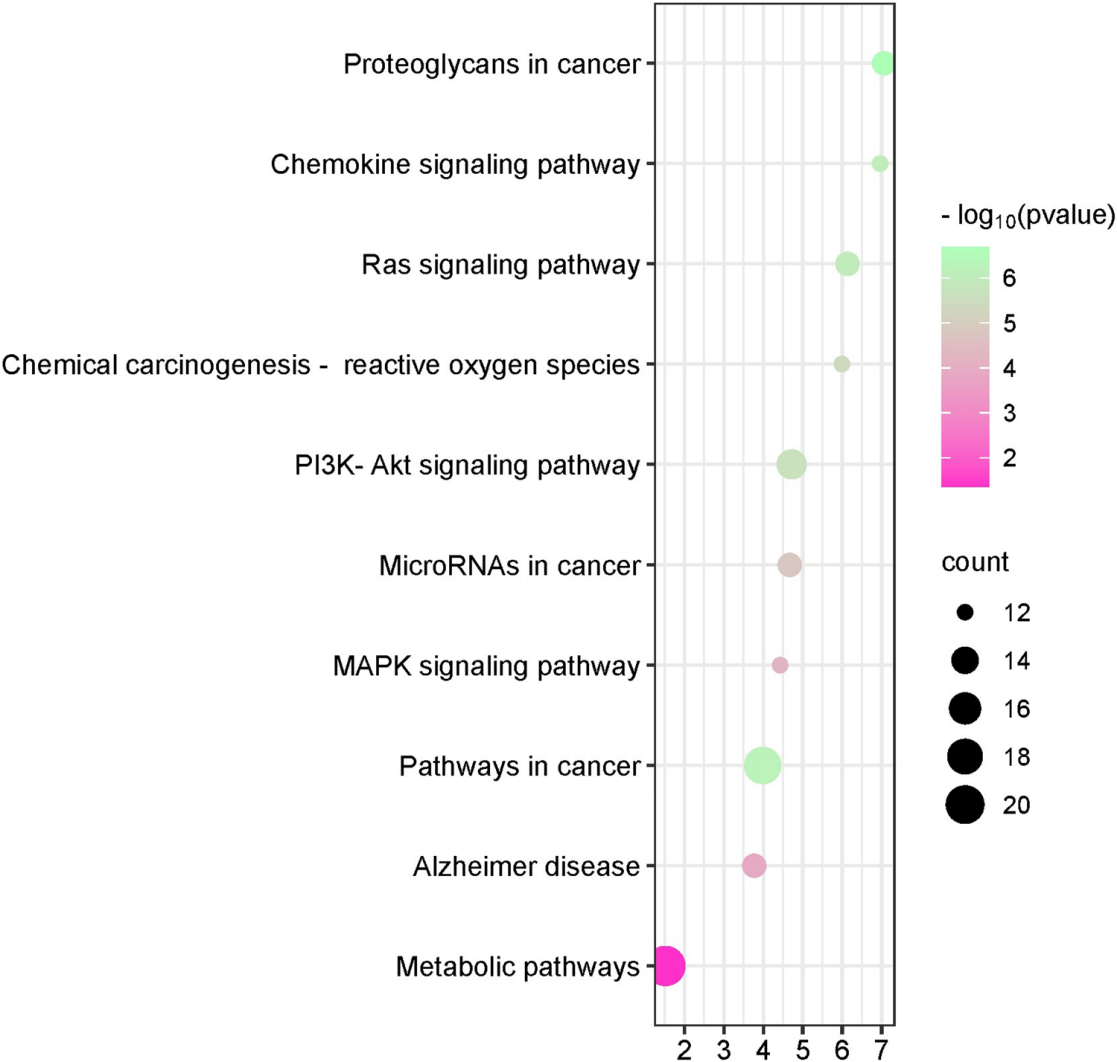
The KEGG pathway of the top targets of hub-genes.

A total of 476 GO items were obtained from the DAVID database which included 323 BP items, 54 CC items, and 99 MF items. We further selected the top 10 BP, CC, and MF catalogs for visualization after filtering them based on the p-value (less than 0.05) and highest count. In the histogram, the ordinary axis represents the degree of enrichment. According to our study results of BP, the function of Q, Q3OG in AD mainly focused on positive regulation of MAPK activity, peptidyl-serine phosphorylation, protein phosphorylation, negative regulation of apoptotic process, positive regulation of cell proliferation, apoptotic process, G-protein coupled receptor signaling pathway, signal transduction, regulation of transcription from RNA polymerase II promoter. The MF items mainly included protein kinase activity, protein serine/ threonine kinase activity, tyrosine kinase activity, protein homodimerization activity, enzyme binding, kinase activity, zinc ion binding, ATP binding etc. the abundant GO functions can also contribute to explaining to a certain extent that Q, Q3OG can be used to treat AD. KEGG pathway enrichment analysis showed Q, Q3OG was mainly involved in 141 signaling pathways. The top 10 enriched pathways were visualized by a bubble chart, the bubble size and the p value represent the extent of enrichment. The main pathways of enrichment included metabolic pathway, Alzheimer disease, MAPK signaling pathway, PI3K-Akt signaling pathway, Ras signaling pathway, proteoglycans in cancer, chemokine signaling pathway, ROS signaling. The Biological process, Cellular Component Molecular Function pathways of the hub-genes are illustrated in Figure 3. The KEGG pathway of the top targets of hub-genes were illustrated in Figure 4. The Biological pathway data is given in supplementary file 4. The Cellular component data is given in supplementary file-5. The molecular function data is given in supplementary file-6. The KEGG-Pathway data is given in supplementary file-7.

### PPI network analysis

**Figure 5:**
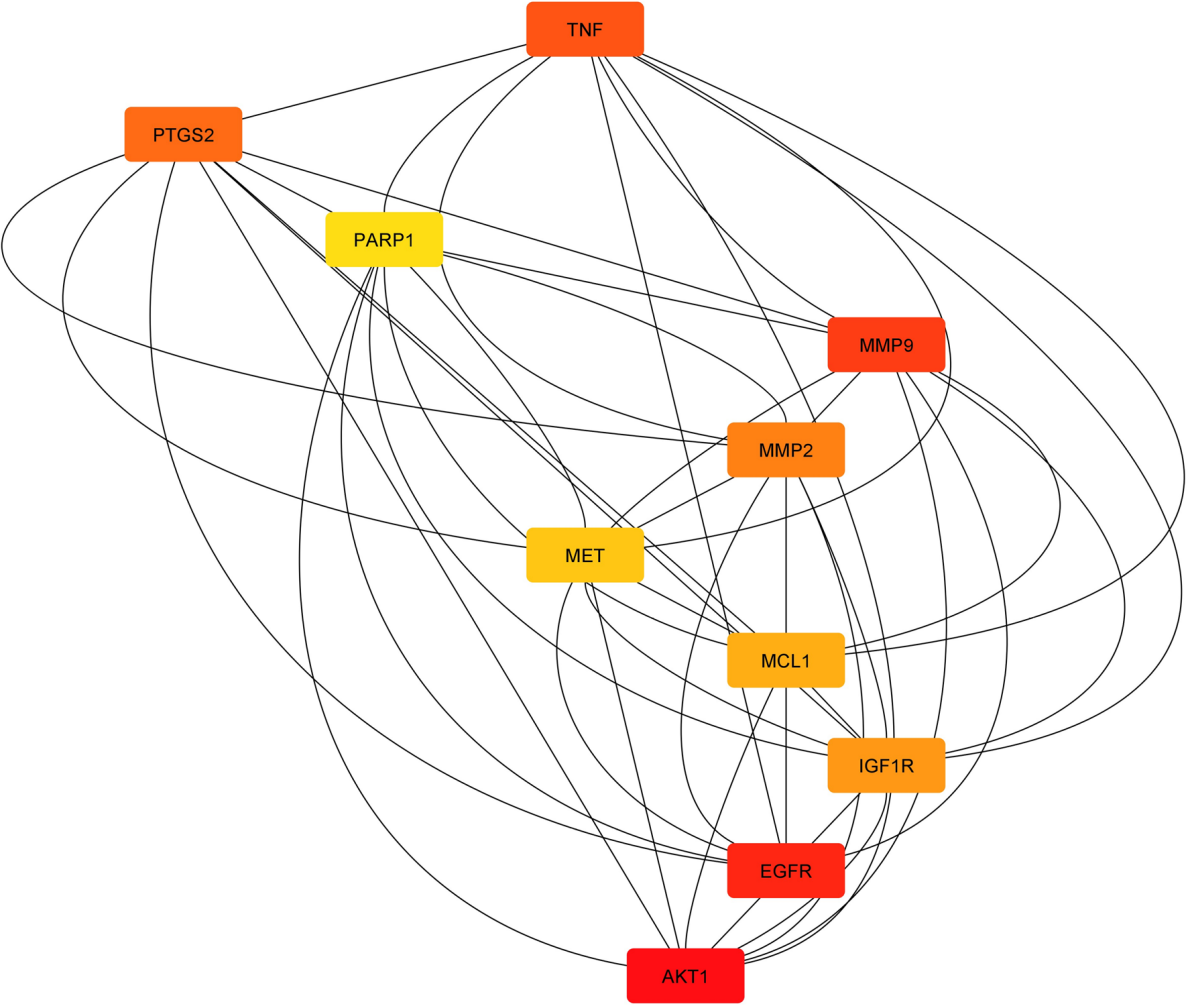
Network analysis of top-ranked targets. (Red color indicates top order -Highly interactive; Orange color indicates -moderate interactive; yellow color indicates -mild interactive).

A total of 77 hub genes were imported into STRING database for PPI network analysis, they cytoscape software was used to visualize and analyze the network by calculating the degree and the higher the degree, important the role of the gene in the network and by using the Cyto Hubba plug in. The top 10 core targets were EGFR, TNF, MMP9, AKT1, PARP1, MET, MCL1, IGF1R, PTGS2, and MMP2. The network analysis of top-ranked targets are depicted in Figure 5. The top ranked targets is given in supplementary file-8.

### Molecular docking analysis

**Figure 6:**
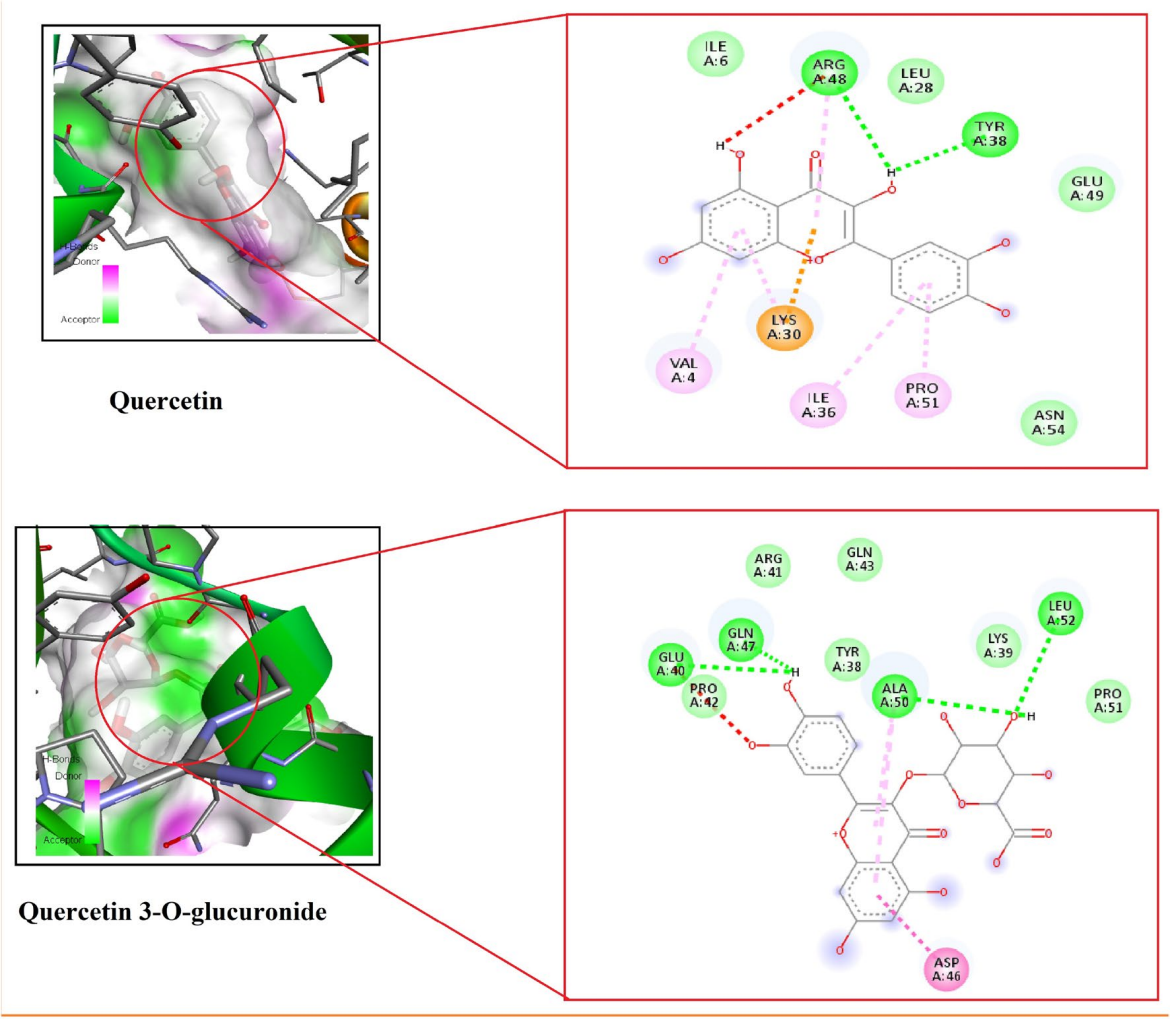
Molecular docking image of Quercetin and Quercetin-3-O-glucuronide.

The molecular docking analysis was performed for Q and Q3OG towards PDB:1UNQ. The binding score of Q was found to be -6.2 kcal/mol with VAL4I, LE6, LEU28 LYS30 THR34 ILE36, TYR38, ARG48, GLU49 ALA50 PRO51, ASN54 amino acid residues. The binding score of Q3OG was found to be -6.58 kcal/mol with GLY37 TYR38 LYS39 GLU40 ARG41 PRO42 GLN43 ASP46 GLN47 ARG48 ALA50 PRO51 LEU52 amino acid residues. The docking score of Q and Q3OG seems to be similar. The activity shows that both of bioactive compounds tends to act on the active sites in efficient manner. The molecular docking score and amino acid residue of Q and Q3OG is given in Table 3. The docking images of Q and Q3OG is illustrated in Fig. 6.

**Table 3:** Molecular docking report and amino acid residues of Q and Q3OG towards PDB:1UNQ. Significant values are in bold.

Compound	PDB:1UNQ	Amino acid residues
Quercetin	− 6.2 kcal/mol	VAL4I, LE6, LEU28 LYS30 THR34 ILE36, TYR38, ARG48, GLU49 ALA50 PRO51, ASN54
Quercetin 3-O-glucuronide	**− 6.58 kcal/mol**	GLY37 TYR38 LYS39 GLU40 ARG41 PRO42 GLN43 ASP46 GLN47 ARG48 ALA50 PRO51 LEU52

### Molecular dynamic simulations of Protein kinase B, in complex with Quercetin and Quercetin-3-O-glucuronide

**Figure 7:**
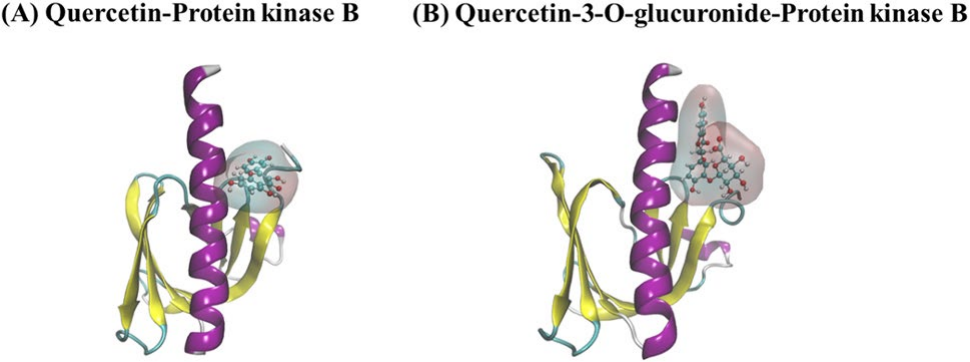
Graphical representation of protein–ligand complexes: (**A**) Quercetin-Protein kinase B and (**B**) Quercetin-3-O-glucuronide-Protein kinase B, where protein is shown in cartoon representation and the ligand is shown in CPK representation with transparent surface.

In order to understand the conformational changes and evaluate the binding of Quercetin and Quercetin-3-O-glucuronide against Protein kinase B (PDB ID: 1UNQ) we have carried out MD simulations for a period of 100 ns for two models namely, Quercetin-Protein kinase B and Quercetin-3-O-glucuronide-Protein kinase B (Figure 7). Their simulations were evaluated using various statistical parameters including Root-Mean-Square-Deviation (RMSD), Root-Mean-Square-Fluctuation (RMSF), h-bond interactions, and its %occupancies over time.

### RMSD analysis

**Figure 8:**
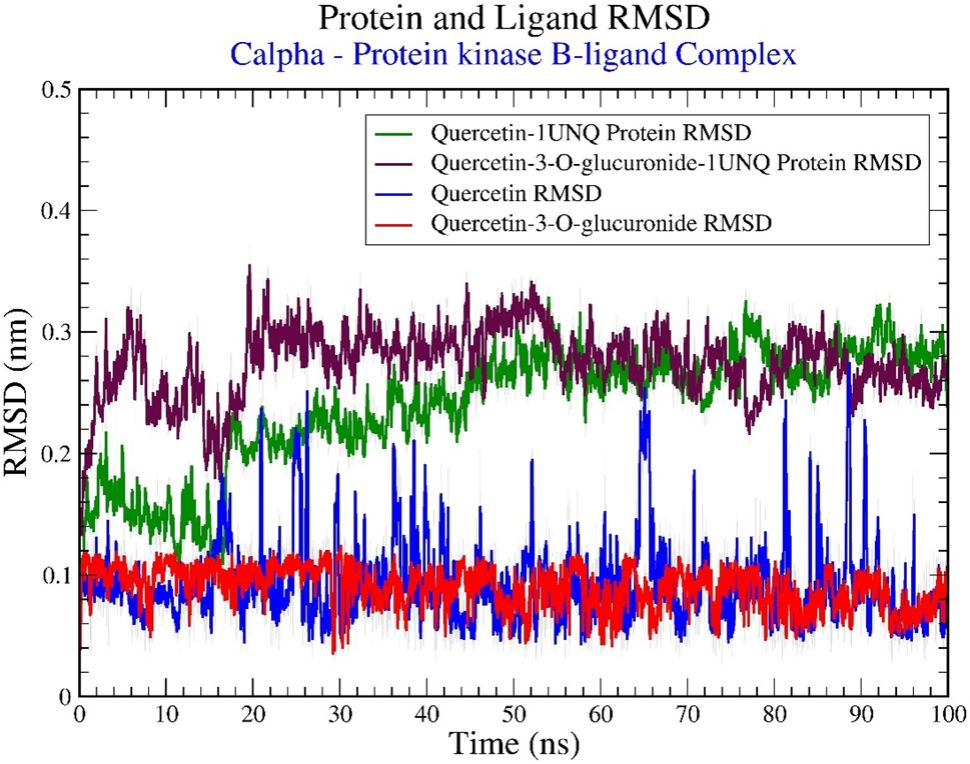
Graphical representation of the plots showing RMSD (nm) versus time (100 ns) for (**A**) Quercetin-1UNQ Protein RMSD (green in color), (**B**) Quercetin-3-O-glucuronide-1UNQ Protein RMSD (maroon in color), (**C**) Quercetin Ligand RMSD (blue in color) and (**D**) Quercetin-3-O-glucuronide Ligand RMSD (red in color).

Analyzing the RMSD can give insights into any structural conformation that protein and ligand undergoes during the simulation. The multiplot for RMSD versus time for 2 simulations is shown in Figure 8. Both complexes have attained a plateau in RMSD values (of around 0.25 nm), indicating that both the ligand-protein complexes were stable during the simulation. Specifically, the ligand Quercetin-3-O-glucuronide displayed a significantly stable deviation of 0.1 nm throughout the simulation. Contrary, the RMSD value of Quercetin has deviated between 0.25 nm to 0.08 nm. But overall, both the ligands have shown RMSD values of lower magnitude which indicated their capability in binding Protein kinase B.

### RMSF analysis

**Figure 9:**
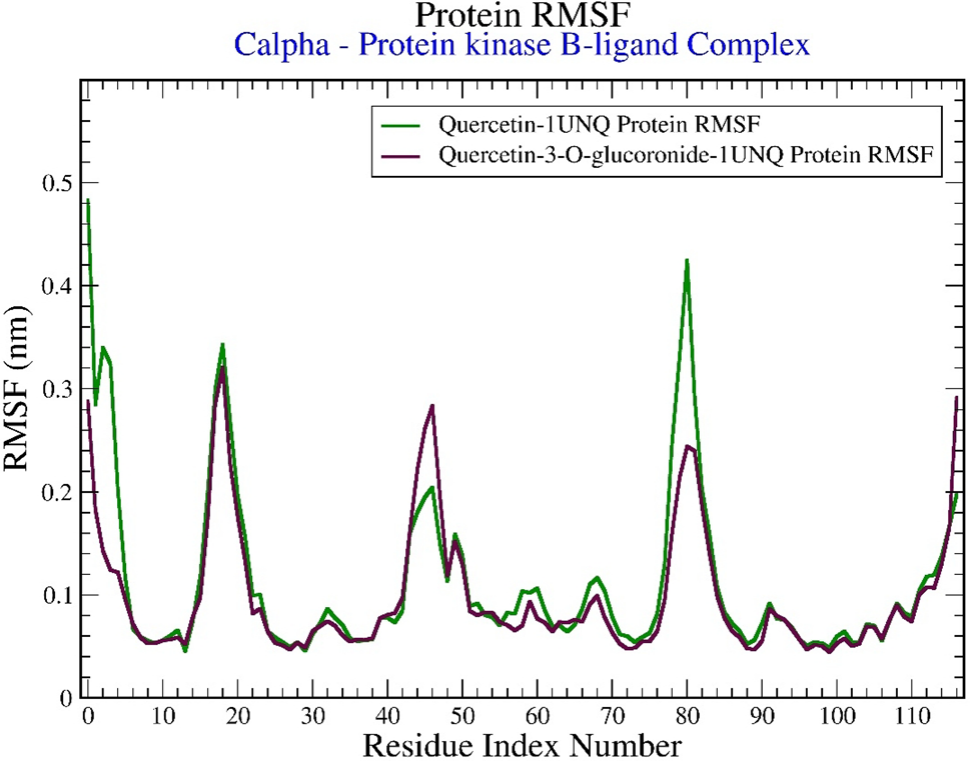
Graphical representation of the plots showing the protein RMSF (nm) versus residue index number of protein for (**A**) Quercetin-Protein kinase B (green in color) and (**B**) Quercetin-3-O-glucuronide-Protein kinase B (maroon in color) complex.

The Protein-RMSF is useful for characterizing local changes along the protein chain. The multiplot for protein-RMSF (nm) versus residue number index is shown in Figure 9. Notably, the plot describes fluctuation of less than 0.45 nm for most of the protein residues*.* However, the loop region of ARG15-TRP22 and beta sheet region of ILE175-VAL90 have displayed fluctuation of 0.35-0.45 nm. But as the residues of this region does not contribute the binding site of the ligand, thus the fluctuations of this region have not affected the ligand binding.

### H-bond interaction

**Figure 10:**
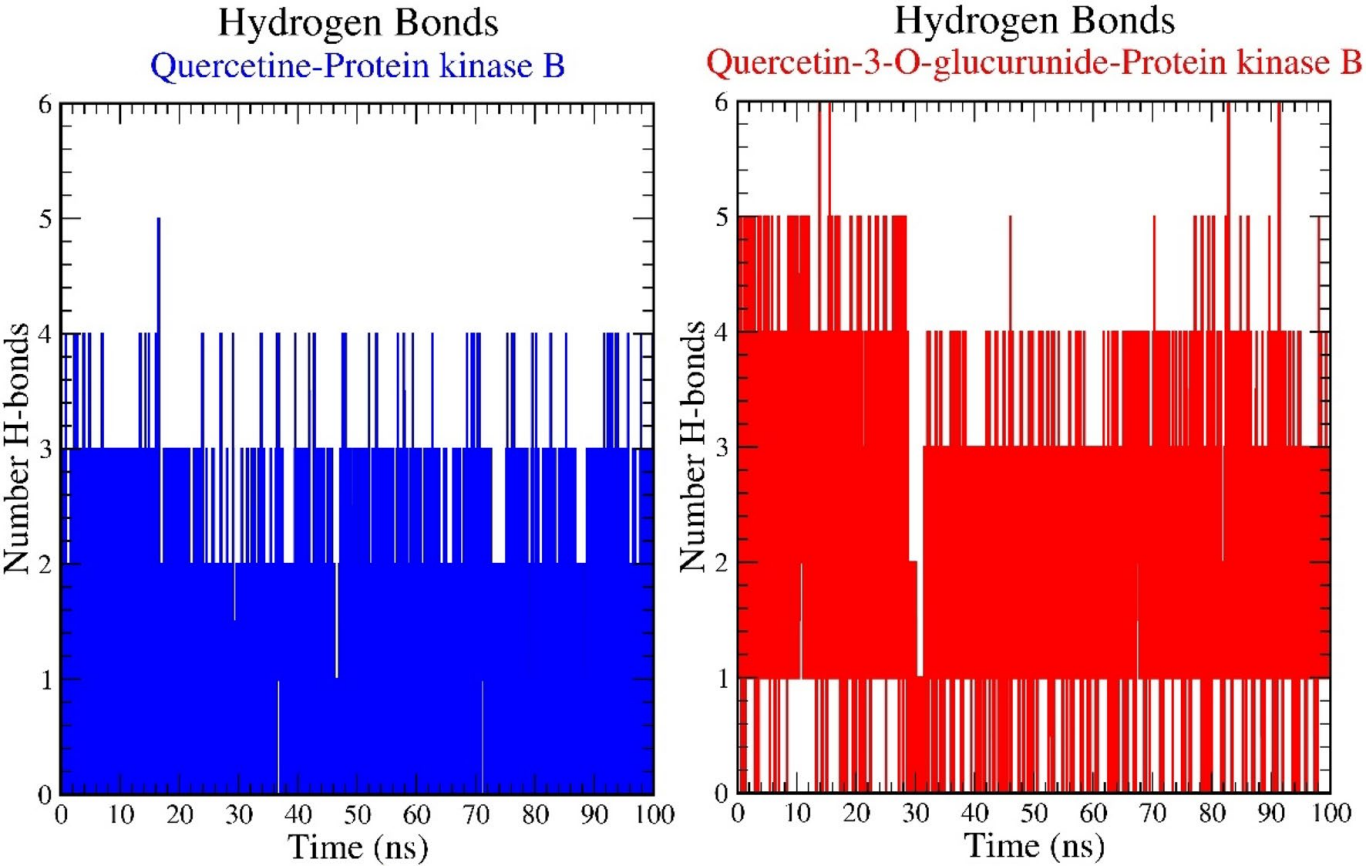
Pictorial representation of the number of h-bond contacts formed by ligands, (**A**) Quercetin and (**B**) Quercetin-3-O-glucuronide in complex with Protein kinase B (PDB ID: 1UNQ).

**Figure 11:**
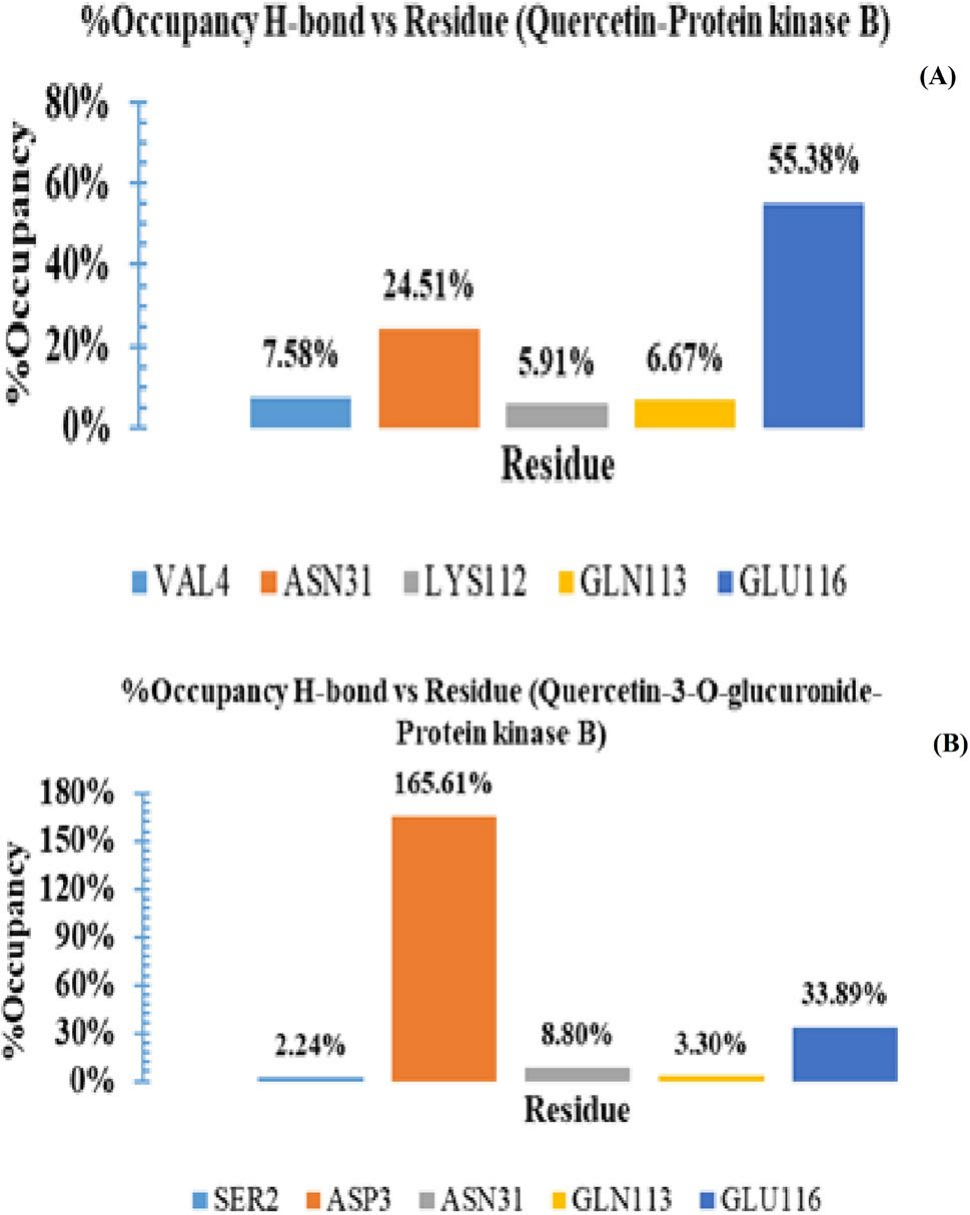
Histogram representation of %occupancies of the h-bond protein–ligand contacts of (**A**) Quercetin and (**B**) Quercetin-3-O-glucuronide in complex with Protein kinase B.

Molecular interactions, particularly the h-bond interactions are distance and angle depend and liable to disruption under dynamic conditions. Herein, we have analyzed the h-bond interactions for both complexes. The plot for the number of hydrogen bonds vs. time is shown in Figure 10. From the plot, it was observed that Quercetin-3-O-glucuronide displayed comparatively better h-bond contacts during the simulation. The Figure 11 shows the Histogram representation of %occupancies of the h-bond protein-ligand contacts of (A) Quercetin and (B) Quercetin-3-O-glucuronide in complex with Protein kinase B. To access the residues involved in such interactions and their stabilities, the %occupancies vs. the residues were also calculated.

## Discussion

Bioactive compound-based novel chemical entities have recently been investigated to increase efficacy and avoid the toxicities connected with the typical treatments of neurodegenerative illnesses. As a result, preclinical investigations have shown that a variety of plant-derived drugs and their extracts that target oxidative stress, neuroinflammation, and mitochondrial dysfunction have significant potential. Alzheimer's disease (AD) is a brain disorder and stands as the most prevalent form of dementia, which disrupts daily functioning by impacting cognitive abilities. The primary risk factor associated with AD is advancing age. Those affected by AD are typically individuals aged 65 and older, and this significantly compromises their overall health and quality of life^28^. Current strategies geared towards AD treatment and the clinical evaluation of drugs primarily address its symptoms, lacking a definitive cure for the condition. There exists a necessity for in-depth exploration to discover promising drug compounds and targets that can mitigate the risk of cognitive decline linked to AD^29^. A further hurdle in AD treatment lies in the effective delivery and precise targeting of pertinent molecules to the brain, impeded by the protective barriers of the central nervous system. Since the brain is the intended site of action, a prominent challenge is surmounting the blood-brain barrier (BBB) ^30,31^. Q is a potent anti-oxidant flavonoid and more specifically a flavonol. It is also known for its anti-inflammatory, anti-hypertensive, and vasodilator effects^32^. Quercetin and Q3OG are bioactive compounds with the molecular weight of 302.23 g/mol and 480.4 g/mol respectively. Q is having 7 H-bond acceptors and Q3OG is having 13 H-bond acceptors. The Blood Brain Barrier(BBB) penetration of Q is -1.098(Numeric (log BB) and Q3OG -1.322 (Numeric (log BB)^33^. The Q3OG seems to be toxic (class %) when compared with Q (class 3). The toxicity scale justification is given in Table 4. The pharmacokinetic parameters are crucial for understanding how a drug behaves within the body and how it may interact with other drugs or physiological processes. Here's why they are important:

**Table 4:** Toxicity profile of Q and Q3OG from Protox-II online database (https://tox-new.charite.de/).

Quercetin	
Quercetin 3-O-glucuronide	

Bioavailability: The extent and rate of absorption from the gastrointestinal tract determine the systemic exposure of a drug. Differences in GI absorption between Quercetin and Q3OG can influence their dosing regimens and efficacy. Tissue Distribution: BBB permeability affects the ability of a drug to reach its target site within the central nervous system. Drugs with poor BBB permeability may be less effective in treating CNS disorders. P-glycoprotein Substrate Status: P-gp substrates are susceptible to efflux transporters, which can limit their intracellular concentrations and efficacy. This parameter is crucial for predicting drug interactions and optimizing treatment strategies. Cytochrome P450 Interactions: Drug metabolism by CYP enzymes can affect both the efficacy and safety of a drug. Inhibition of specific CYP enzymes, as seen with Quercetin, can lead to altered pharmacokinetics and potentially harmful drug interactions. Q has been found to have a protective action on nerve cells by reducing oxidative stress and calming neuroinflammation. Previous research has investigated Q's potential to counter AD, and the findings indicate that it can hinder the clumping of Aβ protein and the excessive phosphorylation of tau protein. Additionally, it helps bring back acetylcholine levels by preventing the breakdown of acetylcholine due to the AChE enzyme^34^.The transport of cholesterol plays a major role in AD. The metabolism of cholesterol and clearance from the body is processed due to the interplay between liver and the gut microbiome. In late-stage AD the amount of liver produced primary bile acids are low with higher amount of secondary bile acids produced by the gut bacteria. This strongly correlates with decline in cognition or brain changes in AD patients. And also, the age-related gut microbiome imbalance increases the risk of bacteria circulation to the brain increasing the risk of inflammation and brain changes^35^. In the elderly, the gut microbiota displays diminished bacterial diversity, alterations in dominant species, a reduction of advantageous microorganisms, elevated presence of facultative anaerobic bacteria, and a decline in overall short chain fatty acid availability^36^. The disruption in gut microbiota balance caused by the age and AD can impact the metabolism of glycosylated forms of quercetin, such as Q3OG and Q7OG, experiences reduced glycoside hydrolysis, leading to modified absorption and metabolite production^37^. The reduced biological activity due to the disturbed gut microbiome in elderly or AD population because the metabolites formed in the small intestine and liver by biotransformation enzymes include the methylated, sulfo- substituted and glucuronidated forms^38^. This research seeks to investigate the potential of Q3OG, a glucuronidated metabolite of quercetin, as a substitute for Q in alleviating the metabolic constraints observed within the gut microbiome of elderly and Alzheimer's disease (AD) patients. This strategy tackles the issue arising from dysbiosis that emerges when administering Q to these patients. Simultaneously, it examines the biological processes through which both Q and Q3OG regulate neuroinflammation and pathogenic pathways linked to AD via network pharmacology and molecular docking methods. This endeavor enhances our comprehension of utilizing Q3OG as a replacement for Q. The literature survey on Q and Q3OG for its biological activities against AD was conducted. These compounds tend to modulate the neuroinflammation, oxidative stress, clumping of Amyloid-Beta protein, and phosphorylation of tau protein via various mechanisms. One challenge in treating Alzheimer's disease is getting drugs or compounds to effectively cross the blood-brain barrier. Some studies suggest that Q and Q3OG might have the ability to cross this barrier, making them potentially valuable for brain-related conditions^39^. The pharmacokinetic comparison between quercetin and Q3OG suggests that Q3G is the major active component in plasma and tissues after oral administration of quercetin or Q3G in rats. This result can help direct intelligent formulation of products for enhanced and prolonged delivery of flavonoids such as quercetin based on glycoside patterns which is similar to our concept of study^40^. The study outcomes demonstrate the significant role of both Q and Q3OG in the regulation of neuroinflammation. They achieve their anti-AD activity by influencing several genes including AKT1, EGFR, MMP9, TNF, PTGS2, IGFR1, MMP2, MCL1, MET, and PARP1. Additionally, our findings were confirmed by investigating the interaction between compound and target pathways through molecular docking, confirming a robust binding between core compounds and key targets, thus supporting their potential against AD. Molecular docking also reinforced the connection between potent constituents and potential targets. Lastly, ADMET analysis of promising phytoconstituents produced positive results, indicating their suitability as therapeutic candidates. The GO and KEGG enrichment analysis revealed that PI3K-Akt signaling pathway, metabolic pathways, AD related pathways, MAPK Signaling pathway, chemokine signaling pathway are the targets for Q and Q3OG by which they intervene and impede disease progression at a pathophysiological level by influencing the targeted genes of the listed pathways. A study conducted by Lap et al., in the year 2013, in order to investigate the potential of Q3OG as a novel therapy for AD. The study results suggest that Q3OG interferes with initial protein-protein interaction of Aβ1–40 and Aβ1–42 responsible for formation of neurotoxic oligomeric Aβ species and also improved the deficits in hippocampal formation basal synaptic transmission via activation of c-Jun N- terminal kinases and MAPK signaling pathway^41^. Similarly, a study by Mengdai et al., in the year of 2022 shows that Q3OG alleviates neuroinflammation, brain insulin resistance and also apoptosis of brain cells by involving in regulation of metabolic pathways, mainly insulin signaling pathway. The cognition is also improved by Q3OG by ameliorating Aβ accumulation and Tau phosphorylation, restoration of gut microbiota and BDNF levels in the hippocampus^42^. Quercetin and quercetin-3-O-glucuronide facilitated PI3K signaling by positive regulation of serine/tyrosine phosphorylation of insulin receptor substrate-1 (IRS-1) and restoration of downstream Akt/eNOS activation, leading to an increased insulin-mediated NO level in a study conducted by Xu et al., 2013 where the author compared the potential of Q and Q3OG in blocking endothelial insulin resistance through inhibition of ROS associated inflammation via PIK3/Akt signaling pathway inhibition^43^. A study conducted to explore the pro-inflammatory cytokines inhibitory effect of Q3OG by Pei et al., 2023, showed reduction in the activation of inflammasome and also Q3OG exhibited anti-apoptotic effects by inhibition of mitochondrial apoptosis pathway in case of pulmonary injury which suggest that further research may render us insights the mechanism on brain cells^44^. According to Samrat et al., 2017, Q3OG has a favorable effect on neurogenesis through regulating Akt and increasing BDNF protein release, implying Q3OG's therapeutic potential in neurodegenerative illnesses^45^. These results from the above discussed studies, positively supports the results of our study suggesting the use of Q3OG in the place of Q to treat AD via PI3K-Akt inhibition, metabolic pathway, AD related pathways, MAPK Signaling pathway, chemokine signaling pathway. Understanding the pharmacokinetic parameters between primary compounds and secondary metabolites enables clinicians and researchers to predict the in vivo behavior of drugs, anticipate potential drug interactions, and customize treatment protocols to maximize therapeutic efficacy while minimizing adverse effects. This study gives the clear-cut idea about the impact of lead bioactive compound and its metabolites, through this computer aided drug discovery report the Quercetin and its metabolites can be used as bioenhancers like Piperine. Just as Piperine (in Resorin Kit) is utilized as a bioenhancer in certain drug formulations, Quercetin also holds the potential to enhance the efficacy of medications used for chronic illnesses.

## Conclusion

Bioactive compounds have significant direct and indirect beneficial effects on human anatomy. The gut microbes may alter the stability and metabolism profile of the bioactive compound before it even gets absorbed, despite the promising results reported by several researchers towards Herbal formulation. Hence, the present study fills the gap between the multiple active target sites of Q and Q3OG towards Alzheimer's via Network Pharmacology, Molecular docking and dynamic simulation. Therefore, it is reasonable to predict the various targets, which may help fill the knowledge gaps in the disease severity and complexity.

### Supplementary Information

Supplementary Information 1.

Supplementary Information 2.

Supplementary Information 3.

Supplementary Information 4.

Supplementary Information 5.

Supplementary Information 6.

Supplementary Information 7.

Supplementary Information 8.

## Data Availability

The authors declare that the data supporting the findings of this study are available within the paper and its Supplementary Information files.
